# Shot-noise Limited Faraday Rotation Spectroscopy for Detection of Nitric Oxide Isotopes in Breath, Urine, and Blood

**DOI:** 10.1038/srep09096

**Published:** 2015-03-13

**Authors:** Yin Wang, Michal Nikodem, Eric Zhang, Frank Cikach, Jarrod Barnes, Suzy Comhair, Raed A. Dweik, Christina Kao, Gerard Wysocki

**Affiliations:** 1 Electrical Engineering Department, Princeton University, Princeton, NJ 08540, USA; 2 Department of Pathobiology/Lerner Research Institute, Cleveland Clinic, Cleveland, OH 44195, USA; 3 Pulmonary and Critical Care Medicine/Respiratory Institute, Cleveland Clinic, Cleveland, OH 44195, USA; 4 Department of Medicine, Baylor College of Medicine, Houston, TX 77030, USA

## Abstract

Measurement of NO and/or its metabolites in the various body compartments has transformed our understanding of biology. The inability of the current NO measurement methods to account for naturally occurring and experimental NO isotopes, however, has prevented the scientific community from fully understating NO metabolism in vivo. Here we present a mid-IR Faraday rotation spectrometer (FRS) for detection of NO isotopes. The instrument utilizes a novel dual modulation/demodulation (DM) FRS method which exhibits noise performance at only 2 times the fundamental quantum shot-noise level and provides the record sensitivity in its class. This is achieved with a system that is fully autonomous, robust, transportable, and does not require cryogenic cooling. The DM-FRS enables continuous monitoring of nitric oxide isotopes with the detection limits of 3.72 ppbv/Hz^1/2^ to^14^NO and 0.53 ppbv/Hz^1/2^ to^15^NO using only 45 cm active optical path. This DM-FRS measurement method can be used to improve the performance of conventional FRS sensors targeting other radical species. The feasibility of the instrument to perform measurements relevant to studies of NO metabolism in humans is demonstrated.

Nitric oxide (NO) is a highly reactive radical species that plays an important role in many chemical processes ranging from atmospheric chemistry (e.g. ground ozone formation^1^) to bio-medical science (e.g. as an inflammatory marker^2^). Significance of the research conducted by Robert F. Furchgott, Louis J. Ignarro and Ferid Murad, who contributed to recognition of the NO as a signaling molecule in the cardiovascular system has been recognized with the Nobel Prize in medicine and physiology in 1998. Due to its high reactivity NO occurs at very low concentrations. In bio-medical applications such as noninvasive exhaled human breath analysis^3^,^4^ or in studies of the regulation of biological and physiological processes in mammalian cells^5^,^6^,^7^ detection at single ppbv (parts-per-billion in volume, 10^−9^) or even sub-ppbv levels is required. One of the major limitations facing the study of NO metabolism in human health and disease is the inability to easily measure in vivo total body NO production. Many researchers have measured concentrations of gas phase NO in exhaled breath or NO metabolites (mainly nitrate and nitrite) in the blood and urine. In addition, studies utilizing stable isotope tracer techniques have significantly increased understanding of arginine and NO metabolism, but quantitative measurements of rates of NO synthesis determine the rate of transfer of labelled arginine to either citrulline or nitrite/nitrate in blood or urine. To date, no method exists to measure the rate of transfer in exhaled breath; therefore, organ-specific measurements of NO production are not possible^8^,^9^. Furthermore, measurement of labelled citrulline and nitrite/nitrate requires skilled operators and specialized equipment. Clearly, studies of the kinetics of NO metabolism are in need for instrumentation that offers simple operation, robust design and high sensitivity, and which enables monitoring of isotope-labeled NO directly in human breath as well as nitrite/nitrate content in urine and blood samples.

Currently there are several commercially available technologies commonly used for NO detection and most popular include chemiluminescence analyzers^10^ and electro-chemical sensors^11^. However these technologies are not capable of distinguishing between different NO isotopes, which prevents them from being used in advanced bio-medical applications that use isotope labeling techniques (e.g. in metabolic studies). NO isotope measurements are often performed with significantly more complex instrumentation such as mass spectrometry (MS) equipped with resonance enhanced multi-photon ionization (REMPI)^12^. A significant barrier with such a complex systems is a high ownership and maintenance cost as well as the requirement of trained personnel, which limits their use only to highly specialized laboratories.

Optical methods based on laser spectroscopy are also capable of sensitive NO isotope detection and with increasing availability of new turn-key laser sources these methods show potential for instrumentation that can be used by non-experts^13^,^14^.

Specimens studied in bio-medical applications are often quite complex mixtures of various (and often unknown) species, thus the measurement method of choice in addition to high sensitivity must provide the highest chemical selectivity. Not all spectroscopic methods capable of isotopic NO detection can simultaneously fulfill the requirements of high sensitivity and selectivity. For example laser induced fluorescence (LIF)^15^, although very sensitive to NO, exhibits dependence of the measured signal on gas sample composition (primarily due to non-radiative quenching effects). To assure satisfactory accuracy in NO-isotope detection LIF would require sophisticated calibration procedures performed by a qualified operator, which prevents this technique from being broadly applied. A number of mid-IR laser absorption spectroscopy techniques have also been successfully employed to isotopic NO detection^14^. Spectral region around 5.3 μm containing the strongest fundamental ν_2_ ro-vibrational band of NO is usually targeted to assure the highest sensitivity. In addition to that, a variety of sensitivity enhancement schemes such as multi-pass cells^16^ or high finesse cavity enhanced detection^17^,^18^ have been applied to achieve NO detection at sub-ppbv/Hz^1/2^ levels. All absorption based techniques require careful selection of the target transition to minimize spectral interference from other species, but in case of complex gas mixtures a possibility of unintended spectral interference still exist. One of the mid-IR laser spectroscopic techniques successfully used for NO detection that distinguishes itself by providing ultra-high sensitivity and significantly enhanced selectivity is Faraday rotation spectroscopy (FRS).

FRS is sensitive exclusively to paramagnetic species (including NO)^19^,^20^,^21^,^22^,^23^,^24^,^25^, which allows to achieve high selectivity even in the case of spectral interference from diamagnetic species (such as H_2_O or CO_2_) that could be present in the sample gas. Since the probability of finding two paramagnetic species with overlapping spectra is extremely small, an exceptional chemical selectivity can be provided by this method. In biomedical applications, which often deal with complex gas mixtures with unknown composition, this represents a significant advantage. In this paper we will present a new FRS-based method that allows performing measurements of NO isotopes with sensitivities approaching the fundamental quantum limits, while using relatively simple opto-mechanical design that allows construction of reliable instruments suitable for applications in clinical settings.

## FRS detection

In FRS, magnetic field is applied to the sample containing paramagnetic species. A laser beam propagating along the direction of magnetic field is used to probe the sample. Due to Zeeman splitting of the paramagnetic transition the sample exhibits magnetic circular birefringence (MCB, a difference in refractive indices for left-handed, LHCP, and right-handed, RHCP circularly polarized components) and magnetic circular dichroism (MCD, a difference in absorption coefficients for LHCP and RHCP). Due to MCB, when linearly polarized light propagates through the sample it undergoes rotation of its polarization axis (the Faraday Effect) that is proportional to concentration of the target species. The ability of indirect sample modulation via magnetic field allows achieving superior noise suppression, which enables extremely sensitive detection without the need for long optical paths. With short optical paths the MCD effects do not contribute significantly to the measured signals and are usually neglected. Moreover without the need for multi-pass cells or high finesse cavities, FRS systems allow for significantly simpler and more rugged opto-mechanical construction with a potential for further miniaturization.

There are however some limitations in conventional FRS systems that prevent this technology from approaching its fundamental limits of detection (details in Supplemental Information, SI). For example in permanent magnet based FRS (DC-FRS) the ultimate sensitivity is often limited by residual FRS artifacts due to interference fringes^24^ that are not sufficiently suppressed by the balanced detection^26^,^27^,^28^, while in FRS with modulated magnetic field (AC-FRS) high currents needed to produce required magnetic fields cause electro-magnetic interference (EMI) that causes FRS signal offsets^25^ and deteriorates long-term stability of AC-FRS. In this work we have developed a new dual-modulation FRS detection method (DM-FRS) that utilizes both magnetic field as well as laser wavelength modulation to suppress laser noise and other unwanted effects and drifts present in conventional FRS systems^29^. A transportable, robust, cryogenic-free prototype instrument for detection of NO isotopes has been constructed and tested in real clinical conditions to detect^14^NO and^15^NO in human breath, blood and urine samples.

## DM-FRS operation principle

**Figure 1:**
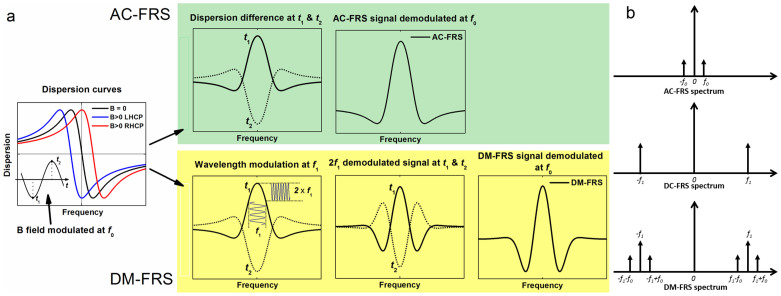
(a) Schematic generation of the DM-FRS signal from the Zeeman-split dispersion line using a two-stage modulation/demodulation process. A process of a conventional AC-FRS signal generation is shown for comparison. (b) Frequency spectra of the AC-, DC-, and DM-FRS signals (DC- and DM-FRS demodulation is assumed to be performed at the first harmonic of *f_1_*).

It is important to realize that the shortcomings of the conventional FRS approaches (both AC-FRS and DC-FRS) are primarily related to the process of signal demodulation that occurs exactly at the harmonics of the applied modulation. This causes unwanted EMI-generated background in AC-FRS or residual signals due to sample absorption or parasitic optical interference fringes in DC-FRS. Moreover low frequency modulation of the magnetic field in AC-FRS makes it difficult to achieve quantum shot-noise limited operation unless high-stability laser drivers and cryogenically cooled detectors are used to lower the technical noise in the low frequency range. In an attempt of suppression of these unwanted effects we have already demonstrated that at high frequencies (>10 kHz) FRS can be performed with sensitivities dominated by the fundamental quantum noise without the need for custom laser drivers (for reduction of laser noise) or cryogenic cooling (for reduction of thermal detector noise). However to achieve that goal a rather complex heterodyne enhanced FRS detection (H-FRS) has been developed^30^, which would be difficult to use outside specialized laboratories. In the new DM-FRS method developed here, a signal-to-noise ratio (SNR) comparable to H-FRS has been achieved with a significantly simpler and robust optical layout, which was the key to enable truly mobile sensor systems. The noise and parasitic effects have been efficiently suppressed through application of dual modulation process: 1) magnetic field modulation occurs at *f_0_*, while 2) the laser wavelength is modulated at the frequency *f_1_* that is much higher than *f_0_* (e.g. for *f_0_* in the single- to sub-kHz range, *f_1_* is selected in the 10–100 kHz range). Within an instant τ (assuming 1/*f_1_* < τ ≪ 1/*f_0_*) the FRS signal can be essentially analyzed using a static magnetic field approximation similar to DC-FRS^24^ demodulated at harmonics of *f*_1_ (at n × *f_1_* where n is an integer number). Over time the magnetic field modulation at *f_0_* will cause the amplitude of the FRS signal at n × *f_1_* to oscillate. Thus the photodetector signal contains different harmonics of the carrier frequency *f*_1_ with the amplitude modulation (AM) sidebands separated by *f*_0_ from the carriers at n *f_1_*. Schematic generation of the DM-FRS spectrum as well as a comparison of the conventional AC- and DC-FRS signals with the DM-FRS signal in the frequency domain are schematically shown in Fig. 1 a and b respectively.

The detection of the DM-FRS signal can be performed through a straightforward AM-demodulation at the carrier frequency that corresponds to the desired harmonic of the laser wavelength modulation (n × *f_1_*). The DM-FRS spectrum demodulated at any of the detected harmonics of *f*_1_ can be calculated as a wavelength modulated version of AC-FRS. A modeling of the Faraday rotation angle and the expected AC-FRS signal has been studied by many groups and is well understood^19^,^22^,^25^,^31^. Thus the DM-FRS signal can be conveniently modeled using the same method as described by G. Wilson^32^ for wavelength modulation spectroscopy (WMS), and the Fourier expansion the n_th_ harmonic spectrum *H*_n_(*x*) can be calculated as:
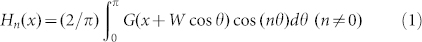
Where *G*(*x*) is a known AC-FRS signal line shape function, *W* is the modulation amplitude with the same unit as the wavelength *x*.

Technically the DM-FRS signal detection can be performed by down-conversion of the signal at any harmonic of *f*_1_ to the baseband using a frequency mixer and by subsequent lock-in detection of the DM-FRS signal at *f*_0_. For practical purposes demodulation at 2*·f*_1_ is performed in this work, because the 2^nd^ harmonic DM-FRS spectrum exhibits maximum at the center of the transition (as shown in Fig. 1a), and its peak value is proportional to the sample concentration. This approach allows performing continuous monitoring of sample concentration with the laser frequency locked to the transition peak. This is possible because unlike conventional AC-FRS the DM-FRS provides true zero-baseline measurement as demonstrated in the following sections of this paper.

## DM-FRS instrument configuration

**Figure 2:**
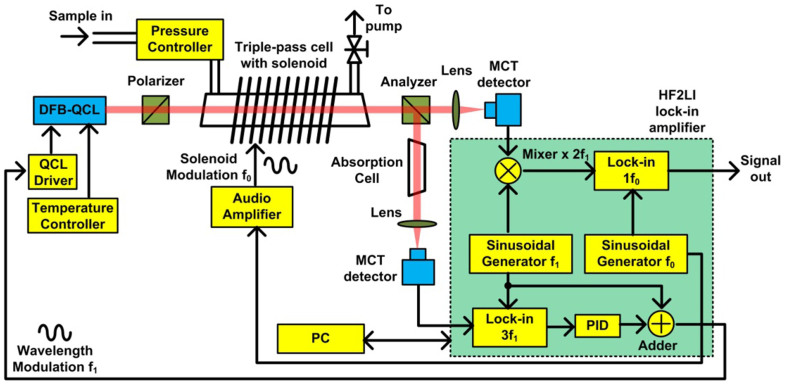
Schematic configuration of a DM-FRS system. The green box indicates all functional blocks for signal generation and processing, which are accomplished by a single digital lock-in amplifier (Zurich Instruments HF2LI).

The configuration of a prototype DM-FRS spectrometer is shown in Fig. 2. The sensor was designed to be able to target both isotopes of NO (^14^NO and^15^NO) and achieve the highest sensitivity to the minor isotope ^15^NO which naturally occurs at much lower concentration (natural abundance of 0.363%). A room temperature DFB-QCL (Alpes Laser S.A.) that gives access to the Q(3/2) transition at 1842.76 cm^−1^ in the ν_3_ fundamental ro-vibrational band of ^15^NO has been used as a light source. This transition yields the strongest FRS signal at low magnetic fields, which guarantees the best SNR for ^15^NO measurements. The same laser source can be tuned (by laser current) to access the P(19/2)e transition at 1842.96 cm^−1^ in the ν_3_ fundamental ro-vibrational band of the major isotope ^14^NO. Due to lower magnetic dipole moment this transition provides ~6× lower sensitivity to ^14^NO; however this is still sufficient for the target application. Such selection of the target transitions proposed earlier by Sabana *et al*^33^ enables detection of both isotopes with a single laser source. The laser beam was collimated with a ZnSe lens with focal length of 1.9 mm and diameter of 4 mm. The laser was operated at 12.5°C (stabilized using Arroyo Instruments 5305 laser temperature controller) with a bias current of ~390 mA, which provided an output power of ~8 mW. The laser current supplied from a low-noise current driver (Wavelength Electronics QCL500) was modulated sinusoidally at *f_1_* = 50 kHz with modulation depth optimized for maximum DM-FRS signal (the frequency of modulation was selected based on the measurement of the noise spectral density in this system, and 50 kHz in combination with 2*·f*_1_ detection was sufficient to effectively avoid 1/f noise component; for details on modulation depth optimization process see SI). Two MgF_2_ Rochon prisms (Foctek RCP5010) were used as the polarizer and the analyzer providing an effective extinction ratio of 1.86 × 10^−4^. To increase the effective path-length without adding alignment difficulty associated with multi-pass cells, a simple triple-pass configuration was formed in the 15-cm-long cell (2 cm in diameter) which resulted in an effective active optical path-length of 45 cm. The cell was surrounded by an air core solenoid (796 turns of 16 AWG copper wire). A resonant circuit was formed by adding a 0.34 μF capacitor in series with the solenoid. A power amplifier (QSC RMX850) was used to drive the resonant circuit at its resonance frequency of *f_o_* = 3.26 kHz. To ensure the maximum signal amplitude the pressure and the magnetic field strength inside the gas cell were optimized to 35 Torr and 158 G respectively (see Refs. 23, 34 for details on pressure/magnetic field optimization). Gas pressure was stabilized using a pressure controller (MKS πPC PC99) and a diaphragm vacuum pump (KNF N920) used to evacuate the sampled gas from the system. After interaction with paramagnetic NO molecules the laser beam propagates through a polarization analyzer (second Rochon prism). The ordinary beam is then focused by an aspheric ZnSe lens onto a thermoelectrically cooled mercury cadmium telluride photodetector (VIGO system PVI-3TE-6) which measures the FRS-modulated light intensity. The photodetected signal is down-converted from the 2^nd^ harmonic of *f*_1_ (2 × 50 kHz) to the baseband using a frequency mixer, and the final DM-FRS signal is lock-in demodulated at *f*_0_ = 3.26 kHz. In the line-locked mode that enables continuous concentration monitoring, the extraordinary beam emerging from the analyzer is recycled and directed through a 5 cm-long reference cell (equipped with CaF_2_ wedged windows) filled with ~1% of NO in N_2_ mixture (the NO content was composed of both ^15^NO and ^14^NO isotopes in amounts assuring approximately equal signal strengths). The 3^rd^ harmonic of the absorption WMS signal demodulated by a lock-in amplifier in the reference branch is used as an error signal for a PID controller that performs feedback controlled locking of the laser wavelength to the center of the target transition. For clinical tests a transportable system was constructed as detailed in the Methods section.

## System characterization

In order to optimize the DM-FRS analyzer performance a full characterization of the noise sources has been carried out. The laser RIN and the photodetector noise equivalent power (NEP) at signal frequency of ~100 kHz were measured to be σ(ω) = 1.78 × 10^−7^ Hz^−1/2^ and NEP(ω) = 1.39 × 10^−12^ W/Hz^1/2^, respectively. An optimum performance (maximum SNR) of FRS spectrometer is achieved at analyzer offset angle that provides laser noise equal to the detector noise^30^. For this DM-FRS system the θ_opt_ is set to 1.8°. A noise-equivalent Faraday rotation angle Θ_NEA_ = 7.11 × 10^−9^ rad/Hz^1/2^ has been determined for this DM-FRS system. Θ_NEA_ is a figure-of-merit that can be conveniently used to compare the performance of different FRS systems^30^.

Another important assessment of the system performance can be performed by comparing the system's Θ_NEA_ to the theoretical limit set by the fundamental quantum noise floor determined by the shot noise of the measured photocurrent. With this assumption the shot-noise-equivalent Faraday rotation angle of Θ_SNEA_ = 3.22 × 10^−9^ rad/Hz^1/2^ has been calculated (see Methods section for details). Based on this evaluation the DM-FRS prototype provides sensitivity that is only 2.21 times worse than the theoretical quantum limit and outperforms by 2.5 times our previous cryogen-free FRS system based on a relatively complex heterodyne-enhanced FRS technique^30^.

**Figure 3:**
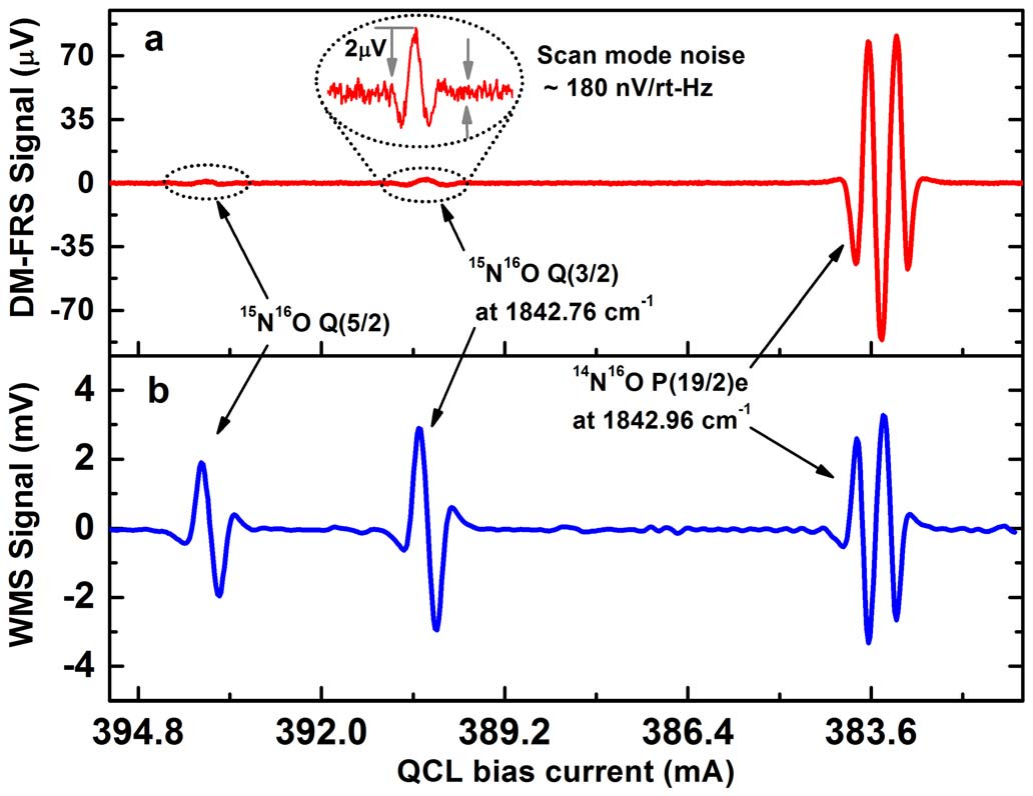
2^nd^ harmonic DM-FRS spectrum acquired in the sample channel (a) and 3^rd^ harmonic WMS signal from absorption cell acquired in the reference channel (b). The laser wavelength was scanned across the spectral region containing the target^15^NO and^14^NO transitions.

Fig. 3a shows a typical DM-FRS spectrum acquired with the laser wavelength scanned across the target transitions of ^14^NO (P(19/2)e doublet) and ^15^NO (Q(3/2) line). A certified mixture of 2 ppmv NO in N_2_ was used in this measurement. Based on the natural isotopic abundance the mixture contains 7.3 ppbv of the minor isotope ^15^NO. The line belonging to the minor isotope is clearly visible in the scan. The conversion factors for the output signal to gas concentration of 25.6 ppbv/μV and 3.65 ppbv/μV were established based on the peak FRS signal values for the ^14^NO (P(19/2)e) and ^15^NO (Q(3/2)) respectively. Seven times higher sensitivity to heavy isotope clearly shows that the performance of the system has been targeted primarily at this isotope through selection of the most optimum optical transition. In a mode of continuous concentration monitoring, only the peak DM-FRS signal is measured and converted into molecular concentration units after prior calibration. Fig. 3b shows the 3^rd^ harmonic (3*f*_1_ = 150 kHz) WMS signal acquired in the reference arm during the same scan. The 3*f* WMS signal, which shows zero-crossing at the center of the transition, is used as an error signal for active line-locking.

**Figure 4:**
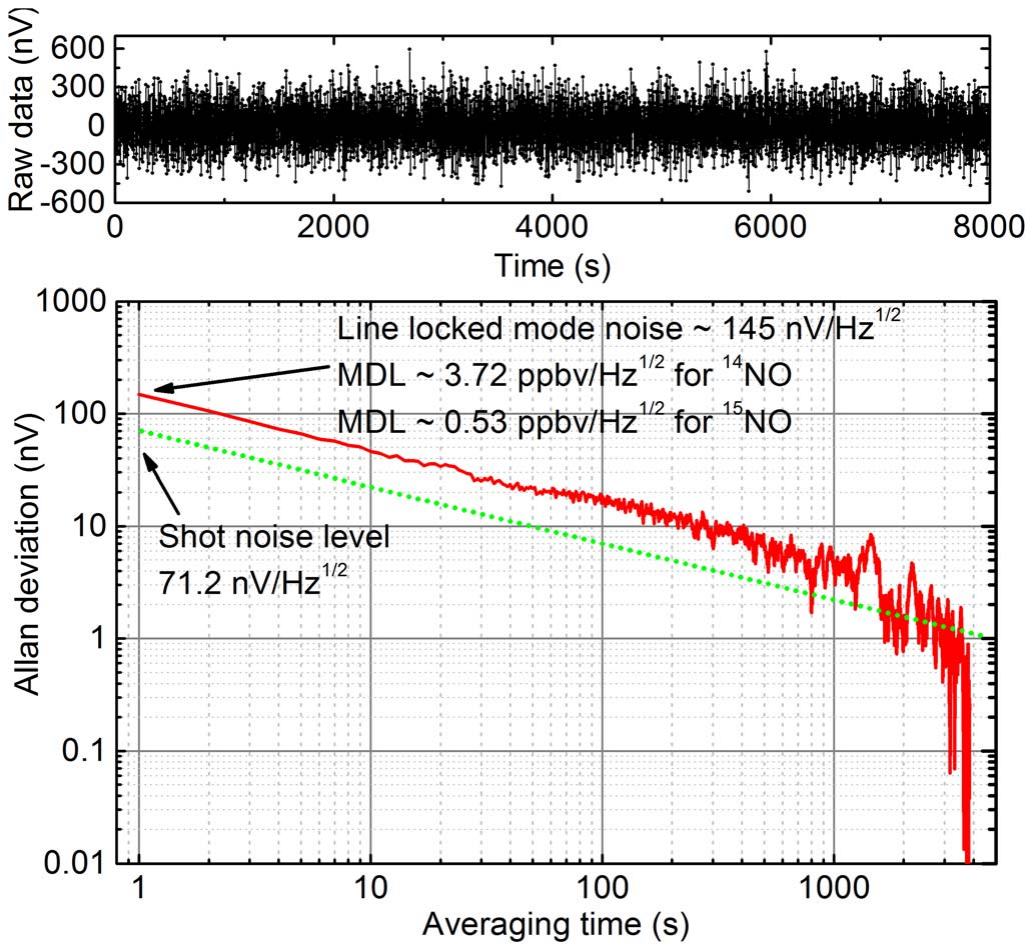
The Allan deviation plot (red) for the measured DM-FRS signal (raw data shown in the upper panel) and for the system shot noise (green dot). The measurement was performed with the laser wavelength locked to the ^15^NO Q(3/2) transition and dry N_2_ was flown through the gas cell.

The system long-term performance has been tested with the laser wavelength locked to the Q(3/2)^15^NO line. To assess the system minimum detection limit a zero gas (dry N_2_) was continuously purged through the sample cell. Fig. 4 shows an Allan deviation plot of the DM-FRS signal acquired with 1 Hz bandwidth and data points sampled in 1 s intervals (raw data shown in the upper panel). Similarly to the spectrum in Fig. 3a the line-locked DM-FRS signal is free from background offset that is usually observed in conventional FRS systems and typically originates from the EMI pickup (in AC-FRS) or parasitic etalons (in DC-FRS). The DM-FRS measurement shows nearly ideal white-noise performance up to integration times of >3000 s. The noise spectral density of 145 nV/Hz^1/2^ measured in the line-locked mode corresponds to a detection limit of 0.53 ppbv/Hz^1/2^ for ^15^NO. Similar performance was observed for ^14^NO. Based on the measured conversion factors this corresponds to the ^14^NO detection limit of 3.72 ppbv/Hz^1/2^. A green dotted line shown in Fig. 4 indicates the Allan plot for the shot-noise level of 71.2 nV/Hz^1/2^ (~2 × lower than the system noise) calculated for the measured photocurrent. This is in very good agreement with the performance estimated from optical measurements of the noise-equivalent Faraday rotation angle which yielded factor of ~2.2 (given the accuracy of experimental determination of laser RIN, or photodetector NEP both values are statistically identical; see Methods for details on measurement techniques). Considering the optimized performance for ^15^NO isotope, the DM-FRS detection limit corresponds to a bandwidth-normalized equivalent fractional absorption of 6.89 × 10^−8^ Hz^−1/2^. This clearly shows that in comparison to the best mid-IR absorption based systems achieving a bandwidth-normalized fractional absorption down to ~2 × 10^−6^ Hz^−1/2^ levels^35^, the DM-FRS system offers ~30 times better sensitivity. This allows performing measurements of paramagnetic species such as NO, NO_2_, O_2_, OH, HO_2_ etc. at ppmv and low ppbv levels without the need for multi-pass absorption cells^35^ or high finesse cavity enhancement^17^,^18^. Therefore the current setup employing an effective optical path of only 45 cm is very practical, enables robust optical configuration, and shows potential for further miniaturization of the instrument.

## Application to bio-medical studies

**Figure 5:**
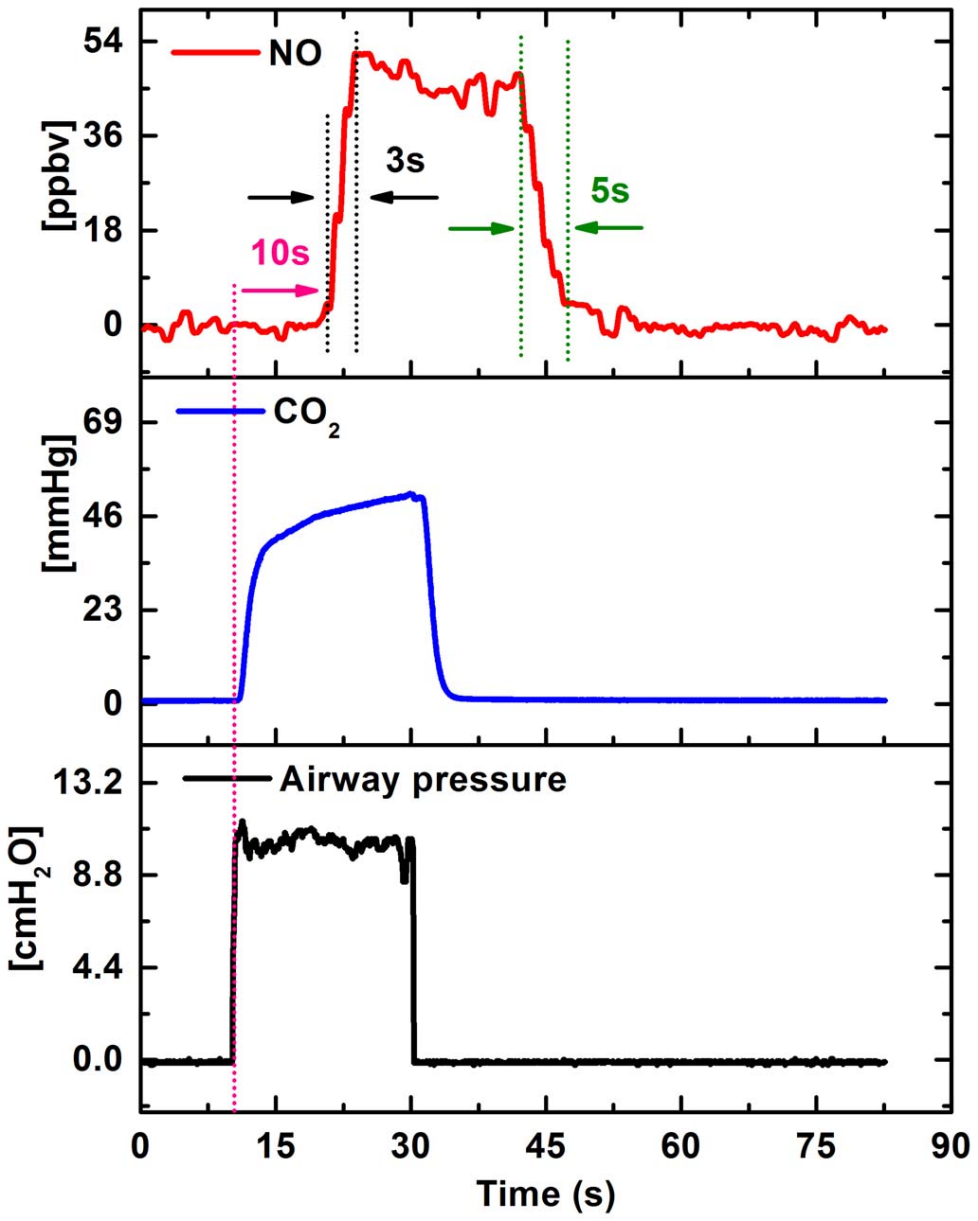
Exhaled ^14^NO (red) concentration acquired using a single breath maneuver. The DM-FRS NO sensor is integrated with a LOCCIONI® single breath sampler which simultaneously measures CO_2_ (blue) and airway pressure (black).

To test the DM-FRS system performance as a bio-medical analyzer of the exhaled NO isotopes we have performed preliminary performance test using a LOCCIONI® single breath sampler integrated with the system. First measurements of the exhaled major isotope ^14^NO (red line) concentration together with the CO_2_ sensor data (blue line) and mouth pressure (black line) provided by the LOCCIONI® breath sampler have been acquired (shown in Fig. 5). This provided characterization of the sampling procedure and time delays associated with the gas flow through the system. There is a 10 second lag time for the NO signal as compared to the CO_2_ and mouth pressure signal measured within the mouth piece of the LOCCIONI® sampler. This has been attributed to the relatively long tubing (~1 m) between the breath sampler and the DM-FRS instrument. The measured NO concentration shows a signal rise-time of ~3 seconds and a clean-up time of ~5 seconds. This confirms that the flow of ~200 mL/min used in this experiment provides the sample cell flush time of <5 s (with cell volume of ~47 mL which at reduced pressure of 35 Torr corresponds to ~2.2 mL of a gas sample at standard pressure), which is sufficient to capture a clear NO concentration plateau (either isotope) during the collection in a single breath maneuver as suggested by the clinical guidelines^36^ and controlled by the LOCCIONI® (built-in heated orifice controls the exhalation flow at 50 mL/s).

In order to enable measurement of nitrite/nitrate in urine and blood samples the DM-FRS was coupled with a commercial unit for chemical conversion of nitrate and nitrite to NO (purge system for Sievers NOA 280i analyzers). This chemical conversion technique has been accepted and reliably used by the bio-medical community for a number of years^37^, and can be used here with DM-FRS gas analyzer to extend its measurement capabilities to liquid samples of urine and/or blood.

**Figure 6:**
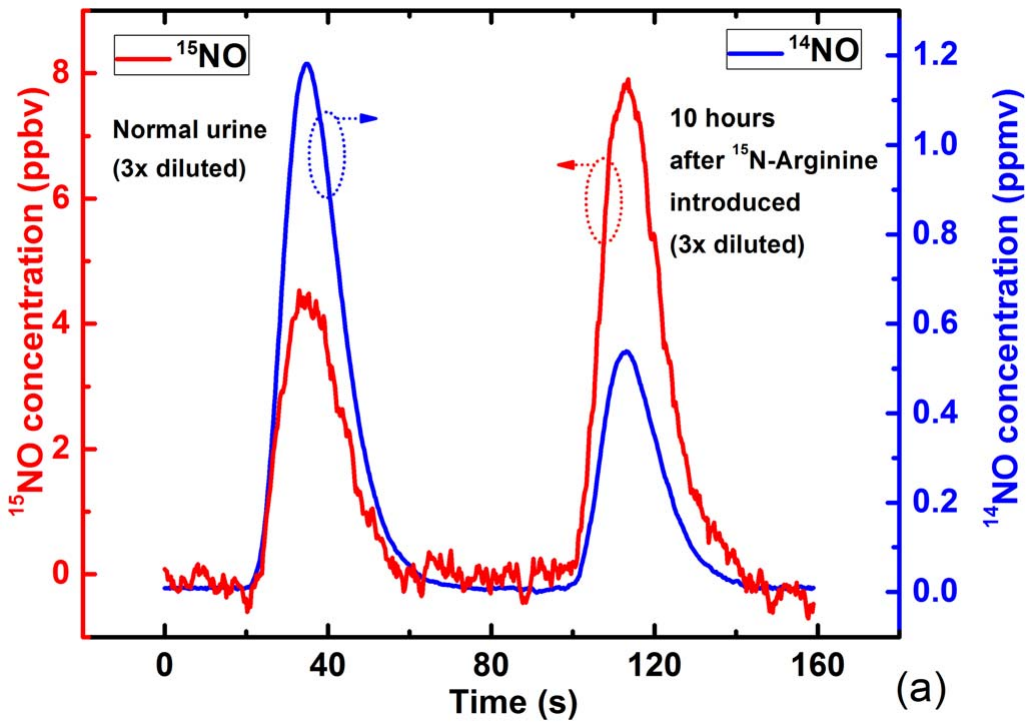
Measurement of ^15^NO (red) and ^14^NO (blue) concentration from a patient's urine samples obtained before (peaks starting at 20 s) and after ^15^N labeled arginine (^15^N-Arginine) was introduced intravenously (peaks starting at 100 s).

The ability of the instrument to detect labeled and unlabeled NO metabolites in liquid samples of biologic specimens was demonstrated using several de-identified blood and urine samples from a metabolic study of arginine metabolism in patients with asthma or pulmonary hypertension and healthy volunteers. After chemical conversion of NO metabolites in liquid samples to the NO isotopes (see Methods for details), NO gas was removed from the samples by bubbling with helium carrier gas and was subsequently detected by the DM-FRS sensor. Because the sensor has been designed to measure one isotope at a time, each sample was divided into two equal portions and each portion was tested for presence of a different NO isotope. Fig. 6 shows an example measurement depicting the concentration peaks of both isotopes when two 3× diluted urine samples were subsequently injected into the reducing agent. In the first sample collected before^15^N_2_-arginine infusion, the peak ^14^NO concentration was ~1.2 ppmv and the peak ^15^NO concentration was ~4 ppbv. The area under the signal peak, which is proportional to the amount of nitrite and nitrate in the sample, is used for calculation of the heavy isotope content. The percentage of the heavy ^15^NO isotope in the sample before arginine infusion is 0.36%, which is in excellent agreement with the natural abundance. For the sample collected after the arginine injection, the peak concentrations of ^15^NO and ^14^NO in the urine sample acquired from the same patient were found to be ~8 ppbv and ~0.54 ppmv respectively. The heavy isotope content increased to 1.41%, clearly indicating the conversion of arginine to NO in the body. In a separate metabolic study of arginine metabolism in women of childbearing age with a similar infusion protocol, breath samples were collected at baseline and between hours 5 and 6 of a 6-hour ^15^N_2_-arginine infusion. For the DM-FRS instrument test we have obtained several de-identified breath samples that were measured directly by introducing them into the FRS sample gas cell. Breath samples were provided in sealed 10 ml test tubes, and due to limited amount of the analyte (see Table 1 caption and Ref. 38), only samples during ^15^N_2_-arginine infusion with elevated ^15^NO could be measured reliably. Table 1 summarizes the results of these measurements and demonstrates the ability of the instrument to detect enriched samples. All breath, urine, and blood samples collected after injection of the ^15^N_2_-arginine show consistently an increased content of heavy isotope. This test presents the ability of this instrument to perform studies of NO metabolism, which are planned as the future stage of this research study.

## Discussion

FRS is based on a very sensitive and chemically selective light-matter interaction principle, but FRS-based instrumentation reported to-date could not fully exploit all its potential. In our previous work we have presented heterodyne-enhanced FRS technique that allowed significant improvement in sensitivity of FRS systems with the total noise of only 3.7 times above quantum shot-noise limit^30^. However, high complexity of the optical set-up prevented that technology from being useful for applications in the environments outside specialized optical laboratories. The new DM-FRS technique presented here is free from these limitations and outperforms H-FRS and other conventional FRS systems in terms of sensitivity as well as in terms of long-term stability and system robustness. DM-FRS allows for truly zero-baseline, large dynamic range, *in-situ* measurements of NO isotopes with sub-ppbv sensitivity. The noise-equivalent polarization rotation angle of 7.11 × 10^-9^ rad/Hz^1/2^ represents over an order of magnitude higher sensitivity than those previously reported systems based on the Faraday Effect^23^,^34^. This translates into the NO detection sensitivity (1σ) of 0.53 ppbv/Hz^1/2^ and 3.72 ppbv/Hz^1/2^ for ^15^NO and ^14^NO, respectively (with 45 cm optical path and magnetic field of 158 G). The total noise observed in this system is only 2 times higher than the quantum shot-noise limit, which has not been achieved before with a non-cryogenic FRS system. The simplicity and robustness of the optical setup and integration of the data acquisition and signal processing electronics allowed a prototype system to become a reliable transportable instrument that could be used in a true clinical environment and enable studies of NO metabolism in human body. In the future this system will be further improved to enable simultaneous concentration monitoring of multiple paramagnetic species (e.g. two NO isotopes measured simultaneously) by using multiple laser sources sharing a common optical path, photodetection, and processing unit. DM-FRS is a unique technique that enables multi-laser platforms with individual encoding/decoding of multiple molecular signals using different carrier frequencies. This makes DM-FRS a very promising approach for a next generation NO isotope spectrometers used in medical studies and potentially other applications (such as environmental monitoring, studies of nitrogen cycle etc.).

## Methods

### System noise characterization

The laser RIN characterization was performed at the target modulation frequency using a noise measurement function provided by the HF2LI lock-in amplifier. In both cases (DM-FRS and AC-FRS) the noise measurement was performed as a function of optical power. A constant laser current of 390 mA and operating temperature of 12.5 °C was used in both measurements and the optical power on the photodetector was varied by rotating a polarizer placed between the laser and the detector. The laser RIN was retrieved from the slope of a linear fit to the noise data as a function of optical power. The RIN measurement are shown in the SI.

The actual quantum shot-noise level is estimated based on the measured photocurrent of ~31.1 μA. The photocurrent shot noise density is calculated as *i*_shot_ = (2e*I*)^1/2^ = 3.16 pA/Hz^1/2^. With 22.5 kΩ transimpedance this corresponds to a shot noise equivalent voltage of 71.2 nV/Hz^1/2^, which is only ~2 times lower than the measured total noise in the system (see Fig. 4).

### Calculation of the theoretical sensitivity limit

To estimate the theoretical sensitivity limit for FRS the equation Θ_shot_ = β·(hν/(2ηP_0_)^1/2^ from Ref. 30 was used. The following parameters of the system have been used: photon energy hν = 3.66 × 10^-20^J, optical power P_0_ = 8 mW, 2^nd^ harmonic signal amplitude loss of β = 1/0.55 = 1.82 and photodetector quantum efficiency of η = 0.73. This results in the shot-noise limited minimum detectible polarization rotation of 3.22 × 10^−9^ rad/Hz^1/2^.

### Transportable system construction

The system was constructed as transportable instrument using a standard 19-inch rack wheel cart (CRUXIAL 352024-L) with the overall size of 60 cm × 45 cm × 100 cm (about half of the size of a conventional AC-FRS sensor system developed in Ref. 23) and was delivered to Cleveland Clinic to test its capabilities to perform measurements of NO isotopes for metabolic studies. To avoid optical misalignment during the transportation or due to temperature drift, high quality kinematic mounts (Polaris) were used, and vibration damping mounts were installed between the optical breadboard and the cart. The signal generation and demodulation, lock-in detection, data acquisition, and PID control was realized using a single digital lock-in amplifier unit (Zurich Instrument HF2LI performing functions marked with a green box in Fig. 2), which allowed for significant reduction of the instrument size and the number of separate table-top test instrumentation units required by the system. A personal computer equipped with custom LabVIEW software was used to control the system (including remote control capability) and provides data storage via USB port. The LabVIEW software provides intuitive user interface with fully automatic functions for system startup, control (including automatic line-locking to selected isotope of NO), calibration and data acquisition. During the 3-month test at the Cleveland Clinic the instrument did not require any optical alignment and all system calibrations performed periodically using certified gas mixtures returned calibration constants within the assessed instrument precision.

### Stable isotope infusion

Tracer infusions were performed in all subjects at the Clinical Research Unit (CRU) at the Cleveland Clinic. Sterile solution of guanidino-^15^N_2_-arginine (Cambridge Isotope Laboratories, Tewksbury, MA, 98+% enriched), was prepared using strict aseptic techniques and were tested for sterility and lack of pyrogens prior to infusion. The protocol was approved by the Institutional Review Board at the Cleveland Clinic and Baylor College of Medicine.

After an 8-hour overnight fast, all participants had an intravenous catheter placed in the antecubital vein for isotope infusions and in a hand vein of the contralateral arm for blood sampling. After a baseline blood sample was obtained, a primed, continuous intravenous infusions of ^15^N_2_-arginine^39^ (prime = 8 μmol·kg^−1^, infusion = 8 μmol·kg^−1^·h^−1^) was started and maintained for 6 hours. Blood samples were obtained every 30 minutes between 4.5 hours and 6 hours (4 samples) of the isotope infusions. Urine and breath was also collected throughout the infusion. De-identified samples were provided to perform this study.

### Measurement of liquid samples

Measurements of NO from liquid samples were performed using nitrate and nitrite reduction apparatus using vanadium (III) chloride in hydrochloric acid chemistry provided by a commercially available purge system for Sievers NOA 280i analyzers (model: CASM 03296-02). The metabolites (mainly nitrate and nitrite) in blood and urine were converted to NO and helium carrier gas was used to transport the produced NO from the reaction solution to the NO DM-FRS sensor. To verify the instrument calibration a series of standard nitrate solutions (with concentrations ranging from 10 to 50 μM) were measured with DM-FRS sensor as well as with Sievers NOA 280i. DM-FRS sensor measurements were consistent with Sievers results within less than <5 μM (the differences were primarily caused by sample preparation and handling). It is worth noting that unlike the chemiluminescence analyzers that require an optimized gas flow rate to assure maximum efficiency of the chemical reaction of NO with ozone, the flow in the FRS cell can be set arbitrarily without affecting the optical measurement. This is especially important for the monitoring of samples with small volumes (such as 10 ml breath samples used in this work).

## Author Contributions

Y.W.,and G.W. invented the DM-FRS method, Y.W., M.N., E.Z. and G.W. developed the instrument, R.A.D. and G.W. planned the instrument feasibility study; C.K. provided samples, Y.W., E.Z., F.C., J.B., S.C., R.A.D. and G.W. performed the study, Y.W., E.Z., F.C., J.B., S.C., C.K., R.A.D. and G.W analyzed data; and Y.W., M.N., C.K., R.A.D. and G.W. wrote the paper.

## Supplementary Material

Supplementary Information Supporting Information

## Figures and Tables

**Table 1:** ^15^NO,^14^NO signals (arbitrary sensor signal units) and their isotopic ratio measured in urine, blood and breath samples collected before and after ^15^N-arginine injection (standard deviations for n measured samples are given for each value indicating significantly smaller distribution of isotopic ratios as compared to individual concentrations of measured isotopes). *Note: Concentrations of exhaled nitric oxide from the lower airways in healthy patients typically vary from 5 to 20 ppbv^38^, which contains ~0.07 ppbv ^15^NO. To reach such sensitivity the DM-FRS sensor needs ~53 s averaging time, which even at the lowest available gas flow rate setting was too long compared to the time required to flow the 10 mL breath samples provided for this study. Therefore it was not possible to measure the^15^NO content in breath samples before the L-[^15^N]2-arginine injection

	Before			After		
	^15^NO	^14^NO	^15^N/^14^N	^15^NO	^14^NO	^15^N/^14^N
Urine n = 13	349.65 ± 229.42	84552 ± 54296	0.41% ± 0.042%	451.65 ± 255.08	38328 ± 18269	1.21% ± 0.33%
Blood n = 13	19.21 ± 10.75	5276.3 ± 2122.8	0.35% ± 0.12%	61.13 ± 27.64	5379.6 ± 2465.9	1.24% ± 0.51%
Breath* n = 3	N/A	N/A	N/A	4.53 ± 0.37	320.7 ± 65.9	1.46% ± 0.22%
