# Supplementary material for: Shot-noise Limited Faraday Rotation Spectroscopy for Detection of Nitric Oxide Isotopes in Breath, Urine, and Blood
Source: Sci Rep. 2015 Mar 13;5:9096. doi: 10.1038/srep09096 (PMC4357895; doi:10.1038/srep09096)
Supplement: Supplementary Information — Supporting Information [file srep09096-s1.pdf]

Supplementary Information

for

Shot-noise Limited Faraday Rotation Spectroscopy for Detection of Nitric Oxide Isotopes in Breath, Urine, and Blood

Yin Wang[a,e], Michal Nikodem[a,f], Eric Zhang[a], Frank Cikach[b], Jarrod Barnes[b], Suzy Comhair[b], Raed A. Dweik[b,c], Christina Kao[d], and Gerard Wysocki[a]

[a]Electrical Engineering Department, Princeton University, Princeton, NJ 08540, USA

[b]Department of Pathobiology/ Lerner Research Institute, Cleveland Clinic, Cleveland, OH 44195, USA

[c]Pulmonary and Critical Care Medicine/Respiratory Institute, Cleveland Clinic, Cleveland, OH 44195, USA

[d]Department of Medicine, Baylor College of Medicine, Houston, TX 77030, USA

[e]Currenlty with Healthy Photon Co. Ltd., 200 East Guoding Road, Shanghai, China

[f]Currenlty with Wrocław Research Centre EIT+, ul. Stabłowicka 147, 54-066 Wrocław, Poland

Review of conventional FRS techniques

In a conventional FRS system a polarizer is placed before the sample cell to establish a well-defined polarization of light and a nearly crossed analyzer is placed after the sample to convert Faraday rotation to intensity changes that can be detected by a conventional photodetector. This method is usually referred to as a 90-degrees method and the nearly crossed analyzer provides effective suppression of the laser relative intensity noise (RIN) [1]. Alternatively an analyzer that gives convenient access to both polarization components (e.g. Wollaston prism) can be set at 45-degrees and two photodetectors can be used to perform balanced detection of the FRS signals (so called 45-degrees method). In 45-degrees FRS method the laser RIN and residual MCD effects that occur as common-mode on both detector elements can be suppressed through electronic subtraction of the photocurrents (balanced detection). The same operation retrieves the FRS signals from both phtodetectors, which are 180o out of phase and thus can be constructively added. A hybrid FRS has also been proposed recently [2], and it takes advantage of both the optical RIN suppression similarly to 90-degrees method and the balanced detection used in 45-degrees FRS method.
Besides various optical arrangements there are two main approaches used to obtain modulation of the FRS signal: an AC-FRS, in which time-varying magnetic field is used to modulate the magneto-optical properties of the sample [1,3], and DC-FRS in which a static magnetic field is used in combination with a wavelength-modulated laser source [4,5]. In both methods the SNR is improved by applying narrowband phase-sensitive lock-in detection at the harmonics of the modulation frequency [4].

Despite the variety of FRS signal retrieval methods, approaching the fundamental limits of detection has been challenging so far. For example in permanent magnet based FRS (DC-FRS) despite efficient reduction of 1/f laser noise through high frequency wavelength modulation, the ultimate sensitivities are often limited by parasitic interference fringes [4] that are not sufficiently suppressed by the balanced detection used in those systems [5-8]. Moreover, in the mid-IR where molecular detection can be performed with the highest sensitivities, availability of commercial balanced photodetectors is very limited and custom devices can be costly. Therefore FRS with modulated magnetic field (AC-FRS) that requires a single photodetector element has been the method of choice in the mid-IR [1,3,9,10]. Selective modulation of magneto-optical properties of the sample used in AC-FRS helps effectively distinguish the spectroscopic signal from parasitic etalon effects. However, high currents needed to produce required magnetic field generate electro-magnetic interference (EMI) that is difficult to control and usually deteriorates long-term stability of AC-FRS systems (electronic pick-ups create fluctuating offsets in the measured signal or cause uncontrolled laser wavelength modulation). Moreover, when driving solenoids with high currents systems must be operated at relatively low frequencies (~ single-kHz), and 1/f noise cannot be efficiently avoided in AC-FRS instruments.

Laser modulation depth optimization

Dependence of the 2nd harmonic DM-FRS signal amplitude on the laser wavelength modulation depth is shown in Fig. S1. For comparison a conventional AC-FRS spectrum the data in Fig. S1 were normalized to the peak AC-FRS signal used as a reference. R shown on the x-axis of Fig. S1 is the ratio of the laser modulation depth *W* to the full width at half maximum (FWHM) of the AC-FRS spectrum measured with the same molecular transition. The DM-FRS signal amplitude reaches maximum at R=1.19. In the reported DM-FRS system, the laser modulation depth was optimized experimentally to yield maximum DM-FRS signal. It should be noted that the maximum signal amplitude expected in the DM-FRS system is a factor of 1.82 smaller than the maximum AC-FRS signal amplitude. This is a direct effect of the secondary modulation process used in DM-FRS. Despite lower signal amplitude, the DM-FRS offers a significant reduction of the noise at high frequencies (1/f noise), which provides a better SNR than AC-FRS.

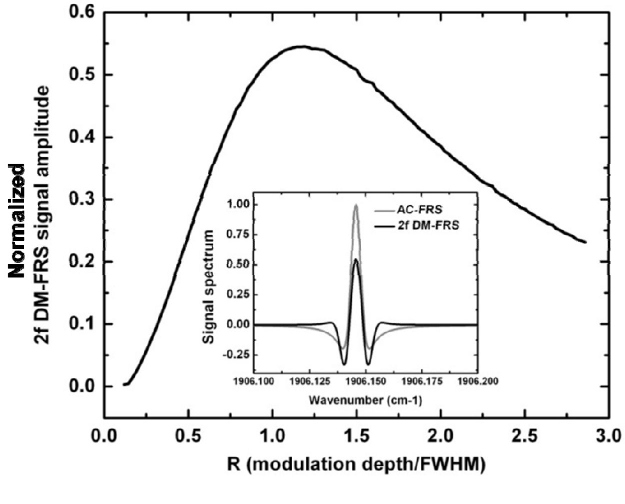

Fig. S1. DM-FRS 2nd harmonic signal amplitude normalized to the peak of AC-FRS signal and plotted as a function of modulation ratio R (R = W/(FWHM of AC-FRS)). The inset shows the spectrum of an AC-FRS system (gray) and the spectrum of a DM-FRS system (black) demodulated at the 2nd harmonic with modulation ratio of R = 1.19.

RIN measurements

The laser RIN characterization was performed at the target modulation frequency using a noise measurement function provided by the HF2LI lock-in amplifier. In both cases (DM-FRS and AC-FRS) the noise measurement was performed as a function of optical power. A constant laser current of 390 mA and operating temperature of 12.5 °C was used in both measurements and the optical power on the photodetector was varied by rotating a polarizer placed between the laser and the detector. The laser RIN was retrieved from the slope of a linear fit to the noise data as a function of optical power received by the photodetector as shown by Fig. S2.

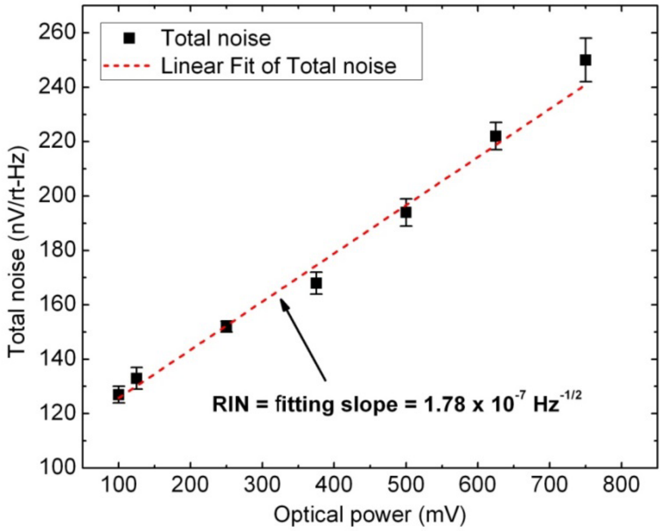

Fig. S2. Linear fit of the measured total noise and optical power indicates the laser RIN at 100 kHz ($2f_1$) where DM-FRS signal is demodulated. The optical power and the total noise are both characterized in terms of the photodetector output signal levels.

REFERENCES:

1 Litfin, G., Pollock, C. R., R. F. Curl, Jr. & Tittel, F. K. Sensitivity enhancement of laser absorption spectroscopy by magnetic rotation effect. *The Journal of Chemical Physics* **72**, 6602-6605 (1980).

2 Zhang, E. J., Brumfield, B. & Wysocki, G. Hybrid Faraday rotation spectrometer for sub-ppm detection of atmospheric O2. *Opt. Expr.* **22**, 15957-15968, doi:10.1364/oe.22.015957 (2014).

3 Lewicki, R., Curl, R. F., Doty, J. H., Tittel, F. K. & Wysocki, G. Ultrasensitive detection of nitric oxide at 5.33 mu m by using external cavity quantum cascade laser-based Faraday rotation spectroscopy. *P Natl Acad Sci USA* **106**, 12587-12592 (2009).

4 So, S. G., Jeng, E. & Wysocki, G. VCSEL based Faraday rotation spectroscopy with a modulated and static magnetic field for trace molecular oxygen detection. *Appl Phys B-Lasers O* **102**, 279-291 (2011).

5 Brumfield, B. & Wysocki, G. Faraday rotation spectroscopy based on permanent magnets for sensitive detection of oxygen at atmospheric conditions. *Optics Express* **20**, 29727-29742 (2012).

6 Hobbs, P. C. D. shot noise limited optical measurement at baseband with noisy lasers. *in Laser Noise, R. Roy, ed.,* **Proc. SPIE 1376**, 216–221 (1991).

7 Hobbs, P. C. D. & Haller, K. L. Double beam laser absorption spectroscopy: shot noise-limited performance at baseband with a novel electronic noise canceller. *in Optical Methods for Ultrasensitive Detection and Analysis: Techniques and Applications, B. L. Fearey, ed.,* **Proc. SPIE 1435**, 298-309 (1991).

8 Durry, G., Pouchet, I., Amarouche, N., Danguy, T. & Megie, G. Shot-noise-limited dual-beam detector for atmospheric trace-gas monitoring with near-infrared diode lasers. *Appl Optics* **39**, 5609-5619 (2000).

9 Ganser, H., Urban, W. & Brown, A. M. The sensitive detection of NO by Faraday modulation spectroscopy with a quantum cascade laser. *Mol Phys* **101**, 545-550 (2003).

10 Murtz, M. *et al.* Magnetic Faraday modulation spectroscopy of the 1-0 band of (NO)-N-14 and (NO)-N-15. *Appl Phys B-Lasers O* **93**, 713-723 (2008).
